# The RHESA-CARE study: an extended baseline survey of the regional myocardial infarction registry of Saxony-Anhalt (RHESA) design and objectives

**DOI:** 10.1186/s12872-016-0336-x

**Published:** 2016-08-17

**Authors:** Katharina Hirsch, Stefanie Bohley, Wilfried Mau, Andrea Schmidt-Pokrzywniak

**Affiliations:** 1 Martin Luther University Halle-Wittenberg, Institute of Medical Epidemiology, Biostatistics, and Informatics, Magdeburger Str. 8, Halle (Saale), D-06097 Germany; 2 Martin Luther University Halle-Wittenberg, Institute of Rehabilitation Medicine, Magdeburger Str. 8, Halle (Saale), D-06097 Germany

**Keywords:** Myocardial infarction, Cohort study, RHESA, Germany

## Abstract

**Background:**

Cardiovascular disease (CVD) is a leading cause of death in Europe. In Germany, a declining mortality rate from acute myocardial infarction (AMI) has been observed in the last decades. Nevertheless, there are large differences between the federal states when looking at the mortality and morbidity of AMI. Saxony-Anhalt is one of the federal states with the highest mortality rates for AMI in Germany. In 2012, the regional myocardial infarction registry of Saxony-Anhalt (RHESA) was established to investigate the individual, infrastructural, and health care factors with respect to an urban (city of Halle) and rural (region of Altmark) population. For detailed observation the RHESA-CARE study was conducted in 2014. RHESA-CARE focuses on the symptoms during infarction, the behaviour of patients while alerting for infarction, the use of rehabilitation possibilities, and long-term care.

**Methods/Design:**

RHESA-CARE is an extended baseline survey of AMI patients registered in RHESA who are aged 25 or more, and inhabitants of the city of Halle (Saale) or the district of Altmark in the federal state of Saxony-Anhalt, Germany. Detailed information was collected on classical and psychosocial cardiovascular risk factors as well as factors of alerting behaviour, first aid, and utilization of medical and rehabilitation services. High data quality is ensured by a detailed system of quality control.

**Discussion:**

RHESA-CARE has the main objective to investigate factors that influence morbidity and mortality rates due to AMI. Another purpose is the comparison of a rural and urban patient population. It provides an opportunity to serve as a base for improvement of patients’ behaviour and health care as well as further research.

## Background

Worldwide, cardiovascular disease (CVD) is a public health problem contributing to 30 % of global mortality (15,616.1 million deaths) and 10 % of the global disease burden [1, 2]. CVD remains also the leading cause of death among Europeans. Over a third of deaths from CVD in the EU are from coronary heart disease (CHD) [3]. One of the five main manifestations of CHD is acute myocardial infarction (AMI). Since 1980 in Germany, the mortality rates of AMI have been declining [4]. However, looking at the occurrence of AMI there are large differences between the federal states of Germany regarding the mortality and morbidity. While in 2012 the age-standardized mortality rate for AMI in Germany was 46 deaths per 100,000 inhabitants, Saxony-Anhalt, a federal state in the eastern part of Germany, has a mortality rate of 67 per 100,000 inhabitants (age-adjusted). However, for Bavaria, a federal state in the southern part of Germany, 47 deaths by AMI per 100,000 inhabitants (age-adjusted) was observed [5]. Saxony-Anhalt is one of the federal states with the highest mortality rates for AMI in Germany. The reasons are still unclear and speculative. Different factors were discussed which could be divided into two fields.

First, individual factors:

- high prevalence of cardiovascular risk factors in Saxony-Anhalt [6, 7],
- low socio-economic level, high unemployment [8–10],
- less use of medical services (ambulant as well as post-hospital) [11],
- alerting behaviour during AMI [12, 13], and
- adherence to medication [14].

Second, structural factors:

- lower doctor density (family doctor and cardiologist),
- low number of first aiders in the population [15],
- guideline care (GLC) ([16, 17]),
- use of german disease management programs (DMP) ([18, 19]),
- insufficient access to rehabilitation units,
- insufficient access to heart training groups, and
- not satisfying guideline medication [16].

To answer the question of which factors influence the mortality rate in Saxony-Anhalt, the regional myocardial infarction registry of Saxony-Anhalt (RHESA) was established in 2012. It investigated some of the factors mentioned above and is described elsewhere [20]. RHESA focuses on the comparison of an urban (city of Halle) and rural (region of Altmark) region. Data collected in RHESA enabled us to assess different levels of quality of health care of patients with AMI (structural, process, and outcome) in these areas. In the registry, we were not able to survey all individual and structural factors mentioned above. Therefore, we conducted an extended baseline survey in 2014. This extension, namely RHESA-CARE, focuses on the symptoms during infarction, the behaviour of patients while alerting for infarction, the use of rehabilitation facilities (multidisciplinary rehabilitation programs, and heart training groups), and long-term care. Furthermore, we plan a follow-up of the MI patients after 2 years of baseline. Here we will examine the occurrence of major adverse cardiac events (MACE) as well as changes in individual cardiovascular risk factors. This paper presents the design and objectives of RHESA-CARE.

The aims of RHESA-CARE are:

- profile the individual factors of AMI patients (Table 1) at baseline and 2 years of follow-up,

**Table 1:** Overview of certain factors in RHESA-CARE split by the time points before/during/after AMI

	Structural factors	Individual factors
Before AMI	Availability of family doctor	Cardiovascular risk factors
	Availability of cardiologist	Use of medical services
		Medication
		Long-term care
During AMI	Availability of first aider	symptoms
		alerting behaviour
After AMI	Availability of rehabilitation	Risk factors
	programmes	Use of rehabilitation programmes
		Use of heart training groups
	Availability of heart training	Medication
	groups	Long-term care
- detect differences in these risk factors between the rural and urban populations of Saxony-Anhalt,
- elucidate reasons for regional variations in structural factors, e.g. availability of a cardiologist, family doctors, and heart training groups,
- examine the influence of individual and structural risk factors and determents like guideline care, heart training groups, and disease management programs on the occurrence of major adverse cardiac events (MACE),
- examine the change in risk profile between baseline and follow-up, and
- quantify the influence of determents on changes in risk profile.

## Methods/Design

### Study design

RHESA-CARE is a extended baseline survey with a planned 2 years follow-up of AMI patients registered in RHESA. RHESA-CARE has been conducted since May 2014 at the Institute of Medical Epidemiology, Biostatistics, and Informatics (IMEBI). It is being funded by the Wilhelm Roux Program of the Faculty of Medicine of the Martin Luther University Halle-Wittenberg.

### Study population

RHESA includes patients with AMI aged 25 years or more. They have to be inhabitants of the city of Halle (Saale) or of the rural district Altmark in the federal state Saxony-Anhalt, Germany [20]. RHESA-CARE comprises all patients from RHESA who have survived AMI and agree with the survey (*N* = 600 per year). All inclusion criteria are shown in Table 2.

**Table 2:** Inclusion criteria of RHESA-CARE patients

Characteristics	
Diagnosis	Survived AMI since April 2014
Age at diagnosis	25 years and older
Residence	City of Halle (Saale)
	District of Altmark
Language	Being capable to complete the interview in German

We include all surviving AMI patients occurring after April 2014. For RHESA-CARE we expect a response of 80 % from all eligible patients. We had to exclude patients who were not capable of completing the interview in German.

### Recruitment of patients and data collection

**Fig. 1:**
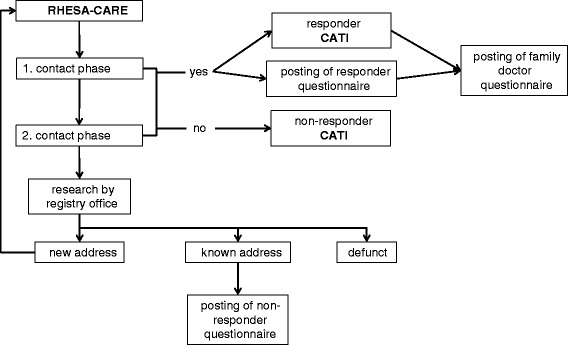
Recruitment scheme of RHESA-CARE

The recruitment includes at least two written invitations and active contact attempts by the study personnel via telephone if the patient does not respond to the written invitations (Fig. 1). Six weeks after discharge from hospital, we contact the patients with a covering letter and a patient information flyer about RHESA-CARE by mail (first contact phase). Patients with a known telephone number are identified and were contacted four working days after the postal contact by telephone. The first contact phase contains 10 phone calls at different times and days. If no contact can be made over at least two weeks, a reminder invitation is mailed to the subject (second contact phase). This phase corresponds to the first phase: covering letter and telephone contact. For those patients without a registered telephone number, two invitation letters with reply-paid envelope are sent. If the patient agrees to the survey, a computer assisted telephone interview (CATI) is fixed, and we submit a confirmation of appointment with additional information. We inform the patients to prepare a list of medication before and after the AMI and hold the address of the family doctor. Responders, who are not able to conduct the CATI (deafness, lack of concentration, or physical disability) obtain a postal self-administered questionnaire. This questionnaire includes the same items as the CATI.

If we were not able to reach the patient we checked address by the respective registry office. Should the address changed, we start the first contact phase again. When it was impossible to reach the patient or the patient does not agree to take part in our study we send them a self-administered non-responder questionnaire.

After the implementation of the CATI, or the self-administered questionnaire, we send a self-administered questionnaire to the family doctor, with a request to answer it, and return copies of the discharge summary from the hospital and rehabilitation unit.

### Questionnaire

After selection of interview items, a pilot study was performed to test the instruments and logistics of data collection. Slight modifications were made before the main phase of RHESA-CARE started in January 2015. The CATI includes classical and psychosocial cardiovascular risk factors as well as factors of alerting behaviour, first aid, and utilization of medical and rehabilitation services. We use highly standardized and validated instruments of data collection which have been applied in several completed or ongoing studies to ensure qualitiy of data and comparability. Questionnaire items were selected and adapted from:

- MONICA/KORA myocardial infarction registry (Cooperative Health Research in the Region of Augsburg) and it‘s postal questionnaire wave in 2011 (KORA-DMP) [19, 21, 22],
- CARLA study (CARdiovascular disease, Living and Ageing in Halle) [23],
- DIOS study (Diagnosis Optimisation Study) [24], and
- IRENA (evaluation of the Intensified Rehabilitation Aftercare Program) [25].

For some special items we developed questions. Details on the sources of the questionnaire modules are listed in Table 3.

**Table 3:** Topics, sub-categories, and sources of the responder questionnaire used in RHESA-CARE

Topic	Sub-categories	Source
Cardiovascular disease	Previous MI, cardiac interventions	Adopted from CARLA
	symptomatic, situation while AMI, stroke, angina pectoris, dyspnoea, fluid retention, atrial fibrillation, blood pressure, cholesterol levels	Adopted from MONICA/KORA and KORA-DMP
	First aid	RHESA-CARE
Diabetes	Type of diabetes, intervention, blood sugar concentration, interval of measurement	Adopted from MONICA/KORA
Utilization of medical services	Consultation rates, medical assistance, patient education (blood pressure, diabetes, haemodilution), DMP programs	Adopted from MONICA/KORA and KORA-DMP
	family doctor	RHESA-CARE
Rehabilitation	Cardiac rehabilitation, heart training groups	RHESA-CARE
Life style	Smoking behaviour	Adopted from BGS98-Questionaire [27]
	BMI, physical activity	Adopted from MONICA/KORA
Medication	Medication before/after AMI, medication use	Adopted from MONICA/KORA, MMAS4 ([28, 29]) also used in MONICA/KORA
Health condition	Health condition	EQ-5D-3L [30] also used in MONIKA/KORA
	Depression	GDS [31, 32]
Care dependency	Care level before/after AMI, use of care service before/after AMI	Adopted from MONICA/KORA
Social status	Socio-economic and employment status	Adopted from CARLA, DIOS [33], and IRENA
	Health insurance	Adopted from MONICA/KORA

We used a non-responder questionnaire to identify possible structural differences between responder and non-responder. This questionnaire is a short version of the responder questionnaire and contains 15 items for cardiovascular disease, rehabilitation, body mass index (BMI), health condition, need for care, and social status. Furthermore, we prepared a questionnaire for the family doctor (FDQ). The FDQ contains the items: blood values, blood pressure, medication before/after AMI, and German disease management program (DMP) [18].

### Quality management

The study was designed to fulfil the recommendations of the German Good Epidemiologic Practices [26]. We include several procedures for quality assurance. Before the main study started, the standard operating procedures (SOPs) were written and a pilot study was conducted. The aim of the pilot study was to test the time plan and the questionnaires.

- After an initial interviewer training course, interviewers were monitored regularly. In addition, we have regular discussions with the interviewers to resolve questions.
- The recruitment progress, given as the number of registered patients, is monitored monthly.
- A plausibility control of the interview data is done monthly and is the basis for regular discussions with the interviewers.
- The self-administered questionnaires are edited visually by the study personnel before the data are entered into the database. Self-administered questionnaires with incomplete information or missing data are marked and questions are prepared for requesting by telephone.
- The FDQ is used to compare the doctors information with that of the patient as well as to complete the data of the patient if necessary.

### Statistical analysis

In the baseline survey we are focused on explorative analysis. Therefore, we plan a descriptive analysis of individual and structural factors. To give an overview of possible differences in urban and rural patients, for example the behaviour of alerting, participation in cardiovascular rehabilitation, and utilization of medical services, we will perform stratified analysis. Furthermore, we focus on gender differences in factors like symptoms of MI, depression, and participation in cardiovascular rehabilitation. To examine the influence of risk factors and structural factors on the occurrence of MACE, we use multiple Cox-regression. For analysis of changing in risk profile we use descriptive statistics. We use SAS®;, Version 9.4 (SAS Institutes, Cary, NC), as well as R®;(Version 3.0.3) for the analysis.

## Discussion

Saxony-Anhalt is one of the federal states of Germany with the highest AMI mortality and morbidity rates. The causes are unclear and need to be surveyed. In previous studies, different factors at an individual patient level were discussed, as well as structural conditions and the quality of process factors.

RHESA-CARE is an extended baseline survey with the aim of a follow-up after 2 years. The main objective of RHESA-CARE is to investigate factors that influence morbidity and mortality rates due to AMI. Another purpose is the comparison of a rural and urban AMI patient population. Because of the selection of standardized interview items applied in other regional studies with the data of RHESA-CARE, a comparison with other registers like MONICA/KORA is possible.

Both RHESA and RHESA-CARE enable us to assess different levels of quality of health care of patients with AMI (structural, process and outcome). In particular, the comparison of rural and urban differences of structural effects and lifestyle components could be described.

The study serves as a base for improvement of patients’ behaviour and health care as well as further research of the named risk factors.

## Abbreviations

AMI, acute myocardial infarction; BMI, body mass index; CARLA, CARdiovascular disease, living and ageing in Halle; CATI, computer assisted telephone interview; MONICA/KORA, myocardial infarction registry of the cooperative health research in the region of Augsburg; CVD, cardiovascular disease; CHD, coronary heart disease; DMP, disease management programme; DIOS, diagnosis optimisation study; DMP, German disease management program; FDQ, family doctor questionnaire; GLC, guideline-care; IMEBI, institute of medical epidemiology, biostatistics, and informatics; IRENA, evaluation of the intensified rehabilitation aftercare program; KORA-DMP, KORA postal questionnaire wave in 2011; MACE, major adverse cardiac events; RHESA, regional myocardial infarction registry Saxony-Anhalt; RHESA-CARE, follow-up of RHESA; SOP, standard operating procedure
